# Can ChatGPT Counter Vaccine Hesitancy? An Evaluation of ChatGPT’s Responses to Simulated Queries from the General Public

**DOI:** 10.3390/healthcare13111269

**Published:** 2025-05-27

**Authors:** Matthew Chung Yi Koh, Jinghao Nicholas Ngiam, Brenda Mae Alferez Salada, Paul Anantharajah Tambyah, Sophia Archuleta, Jolene Ee Ling Oon

**Affiliations:** 1 Division of Infectious Diseases, Department of Medicine, National University Hospital, National University Health System, Singapore 119074, Singapore; matthew.koh@mohh.com.sg (M.C.Y.K.); brenda_mae_alferez@nuhs.edu.sg (B.M.A.S.); mdcpat@nus.edu.sg (P.A.T.); mdcsa@nus.edu.sg (S.A.); jolene_oon@nuhs.edu.sg (J.E.L.O.); 2 Yong Loo Lin School of Medicine, National University of Singapore, Singapore 119077, Singapore; 3 Infectious Diseases Translational Research Programme, Department of Medicine, Yong Loo Lin School of Medicine, National University of Singapore, Singapore 119077, Singapore

**Keywords:** artificial intelligence, ChatGPT, vaccine hesitancy, vaccinations, education

## Abstract

**Background:** Vaccines have eradicated deadly diseases, yet vaccine hesitancy persists, leading to reduced uptake. Some individuals, mistrustful of healthcare providers, may turn to tools like ChatGPT for information. We evaluated ChatGPT’s responses to common vaccine hesitancy questions. **Methods:** Infectious disease physicians compiled 15 frequently encountered questions relating to vaccine hesitancy, focusing on concerns about efficacy, adverse effects, and cultural or religious issues, and submitted them to ChatGPT on 18 October 2023. Two independent physicians assessed the responses for factual accuracy and alignment with recommendations from the U.S. Centers for Disease Control and Prevention’s Advisory Committee on Immunization Practices (ACIP). **Results:** A representative selection of responses is shown. ChatGPT consistently provided fact-based, reassuring responses. For instance, it accurately addressed the benefits of male HPV vaccination; however, it failed to consider ACIP’s age-specific recommendations and individual sexual health factors. While correcting mRNA vaccine misconceptions, it did not mention the availability of non-mRNA COVID-19 vaccines. It also lacked depth in discussing religious objections, deferring users to faith leaders and providers. **Conclusions**: ChatGPT provides generally accurate information and may be a useful adjunct in addressing vaccine hesitancy. With refinement, it could complement public health efforts to improve vaccine confidence and counter misinformation.

## 1. Introduction

Vaccine hesitancy is a phenomenon of global concern, particularly in the context of the recent coronavirus disease 2019 (COVID-19) pandemic [1]. It covers a broad range of factors that contribute to an individual’s behavioural decision to accept a vaccine [2]. From a public health perspective, this leads to excess morbidity and mortality that could have been prevented with improved uptake of vaccines. One study estimated that non-vaccination against COVID-19 contributed towards greater than 200,000 deaths from COVID-19 in the United States alone [3]. Willingness to vaccinate is influenced by several factors that contribute to an individual’s health beliefs. Easy access to reliable and accurate information, as well as trust in these sources, contributes significantly to an individual’s overall knowledge and attitudes towards vaccines [4].

Individuals who are sceptical of data and information provided by scientific publications on vaccinations may turn to alternate sources for information on the safety and efficacy of vaccines [5]. Several unreliable or overly commercial sources provide inaccurate and sensationalised myths and misinformation on vaccinations, which leads to increased vaccine hesitancy and reduced uptake of important life-saving vaccines [6].

In addition to these sources, individuals may also turn to artificial intelligence (AI) natural language processing chatbots such as ChatGPT for information on vaccines [7]. ChatGPT is widely accessible and provides easy access and responses to a wide range of queries posed by individuals. Its use has been studied in several settings, such as medical education [8,9]. Recently, ChatGPT has been examined in various clinical settings, including pre-travel advice and counselling on safe living practices for solid organ transplant recipients [10,11]. However, its role in providing accurate information and addressing concerns pertaining to vaccine hesitancy remains to be understood and examined. Therefore, from the end-user perspective, we sought to assess ChatGPT’s responses to common vaccine hesitancy concerns, in accordance with factual accuracy and alignment with U.S. CDC Advisory Committee on Immunization Practices (ACIP) guidelines.

## 2. Methods

To do so, we instructed ChatGPT (GPT-3.5) to answer a series of frequently asked queries related to commonly used vaccines that may lead to vaccine hesitancy on 18 October 2023. The date cut-off for ChatGPT’s training data was January 2022 at that time. Fifteen questions were drawn from clinician experience in infectious diseases and vaccine counselling and represented commonly encountered concerns among the general public. We divided these questions into the domains of (i) doubting vaccine efficacy, (ii) concerns about adverse effects, and (iii) cultural concerns pertaining to vaccines. In this exploratory analysis, a qualitative assessment of ChatGPT’s responses was performed independently by two physicians (MCYK and JNN). The responses were checked for factual accuracy and appropriateness against information provided by the ACIP guidelines [12].

## 3. Results

ChatGPT answered all the questions posed on vaccine hesitancy (Table A1). An appraisal of specific questions asked, along with summarized responses given by ChatGPT, is shown in Table 1. Overall, we found that ChatGPT was able to provide highly accurate and up-to-date information when asked about specific vaccines. It was able to address vaccine hesitancy in a balanced manner, providing the pros and cons of vaccination as well as encouraging further discussion with a healthcare professional, to allow for shared decision-making between the individual and their provider.

## 4. Discussion

In the domain of vaccine efficacy, the information provided by ChatGPT was accurate. With regards to measles vaccination, ChatGPT was able to accurately highlight that the benefits of vaccination extend beyond the individual and confer herd immunity in the community. In addition, an individual living in low-prevalence settings for measles may travel to higher endemicity areas in their lifetime, or encounter imported cases of measles, giving an important reason for vaccination at an early age to confer lifelong protection. Indeed, with falling rates of measles vaccination, outbreaks have been reported even in historically low-prevalence areas [13]. When discussing the HPV vaccine for men, ChatGPT was able to explain that the vaccine conferred protection against anal, penile, and oropharyngeal cancers, as well as some protection against non-cancerous conditions like genital warts in addition to cervical cancers. Increasing HPV vaccination in men also confers herd immunity, which helps protect women in the community [14].

However, the responses provided by ChatGPT were generic and not tailored to a specific individual. For example, in trying to convince an individual to get vaccinated against measles, it was important to consider and emphasize specific factors such as the likelihood of an individual to travel to areas of higher endemicity or whether the vaccine itself was mandated by regulatory agencies. There were also minor lapses in the factual accuracy on HPV vaccination. The ACIP recommends routine vaccination in men up to 26 years of age [12], while for those between the ages of 27 and 45, the ACIP recommends shared decision-making between the individual and their healthcare providers. This discussion considers an individual’s risk in the form of sexual health and practices as well as individual preferences. ChatGPT did not consider an individual’s age when making a recommendation for HPV vaccination, but it did direct an individual to discuss further with their healthcare provider for more specific and detailed advice.

In the domain of vaccine adverse effects, the advice provided by ChatGPT was also highly accurate. With regards to the influenza vaccine and egg allergies, the advice provided was up-to-date, highlighting that newer influenza vaccines no longer contain egg proteins, which were previously a concern for patients with severe egg allergies [15]. Rather than being dismissive of the concern, ChatGPT gave the historical context for these initial concerns and then provided reassurance with the latest evidence. ChatGPT was also able to firmly dispel other common myths pertaining to vaccines, such as the permanent alteration to deoxyribonucleic acid (DNA) caused by messenger ribonucleic acid (mRNA) vaccines or the association of the measles, mumps, and rubella (MMR) vaccine with the development of autism in children. In these responses, ChatGPT provided a measured view and explained the mechanism by which vaccines work, with a focus on their safety profiles. When asked about specific vaccine concerns, it took the opportunity to encourage and emphasize the importance of vaccination and prompted the individual to discuss further with a healthcare provider.

ChatGPT was also able to address parental concerns with regards to COVID-19 vaccinations, such as the long-term safety of vaccines. It acknowledged the uncertainty of the vaccine’s long-term side effect profile but reassured the individual that the benefits likely outweigh the risks of vaccination, along with the added reassurance that comes from continued monitoring and safeguards in place. However, although the mRNA vaccines are safe in children, ChatGPT did not provide alternatives such as inactivated COVID-19 vaccines, which may still be an option for parents concerned about the mRNA vaccine [16], or sources of information in the event that the individual or parent is eager to find out more about each specific vaccine.

Finally, when addressing cultural concerns with regards to vaccination, ChatGPT was able to provide balanced views. It demonstrated respect for a person’s religious beliefs while simultaneously encouraging open discussion with religious leaders on this topic. It also provided the scientific facts on the benefits of vaccination for the individual to consider, as these are understandably important concerns to address to improve vaccine uptake in certain religious communities across the world [17]. However, given a more specific context, it may be able to address issues specific to an individual’s religion, rather than keeping its responses vague.

When discussing ethical concerns relating to vaccine manufacturing and development processes, ChatGPT’s answers were sensible and measured. It firmly denied the argument that actual human embryos are used in the testing of mRNA vaccines, without going into the details of which vaccines use foetal cell lines [18]. However, it acknowledged that animal testing does occur in the context of vaccine development, with clear explanations that these studies were conducted with care to protect the welfare of the animals involved and cause the least amount of harm or distress. Animal testing is important to determine the safety and efficacy of vaccines, and contributes to the development of vaccines, which would translate to improving human health. Thus, the responses acknowledged an individual’s concerns but also aimed to provide alternative views to address the individual’s vaccine hesitancy. A further important factor pertaining to COVID-19 is the notion of ‘vaccine fatigue’. Where an individual may have initially accepted vaccines, over time, there may be an increasing inertia towards repeated follow-up vaccination. These may reflect changes in health beliefs, and the increasing misinformation propagated pertaining to vaccines [19]. The measured approach that ChatGPT takes in explaining mRNA vaccines may help to allay some of these fears. However, an important limitation is that this model of ChatGPT also did not discuss the possibility of non-mRNA vaccines, which may be considered for individuals who are sceptical about mRNA vaccines. Additionally, ChatGPT did not cite references for its responses, which may be helpful if individuals were keen to check the veracity of the information by referring to the original source.

Overall, our findings highlight the potential role of AI chatbots like ChatGPT as intermediary tools in public health communication, particularly in the context of addressing vaccine hesitancy in individuals who may distrust traditional sources. There remains a tension between the improved accessibility and personalization of responses from the large language model. For example, GPT-3.5 is easy to access at any time and by anyone, but has trade-offs in lacking specificity to the local context and tends to give generic responses. Additionally, it would be important to consider real-world implementation and evaluate the integration of AI tools into clinical or public health workflows. However, this would still need careful human oversight and regulatory guidance. This is because ChatGPT’s responses still remain limited in terms of the specificity of the advice and response provided. The quality of responses depends on how much information is provided by the individual asking the question. For example, unless details of the specific religious or ethical concerns are given, the responses could only remain generic and not tailored to address an individual’s specific beliefs.

We have previously described the use of ChatGPT as an adjunctive tool for pre-travel advice and counselling. Although it helped to enhance the consultation experience for the traveller, it still could not be used in isolation and was merely an adjunctive tool that required follow-up with further counselling from a human healthcare provider [20]. Furthermore, another important consideration is that the large language model algorithms may also be altered without warning, which may inadvertently affect the performance of these models.

## 5. Limitations

Our study examined a single natural language processing chatbot that was the most widely available. Other chatbots that are designed to answer healthcare-related questions more specifically may be able to provide better responses. As this was a preliminary, hypothesis-generating study, we opted for a qualitative assessment by two independent physicians. We did not employ a formal rubric but checked for factual accuracy and alignment with ACIP recommendations. As such, we were not able to meaningfully provide a numerical percentage of ChatGPT’s responses that adequately met pre-defined standards. Future work could formally assess these responses with a structured rubric and measure inter-rater reliability metrics such as Cohen’s kappa. Also, we did not replicate our study at different timepoints, although with more information over time, artificial intelligence chatbots may also improve on the responses provided. Lastly, the topic of vaccine hesitancy is very broad, so the questions examined could not be fully exhaustive. Also, the questions were selected by healthcare professionals, which lends itself to a degree of bias and may not fully reflect the concerns of the general public. Thus, our findings were meant to be hypothesis-generating, demonstrating that with increasing global vaccine hesitancy and mistrust in science, AI chatbots may help to mediate and correct misinformation on vaccines among the general public.

## 6. Conclusions

ChatGPT’s responses to vaccine hesitancy were mostly accurate and may help to correct misinformation and knowledge in individuals who harbour vaccine misconceptions that lead to a reduced willingness to vaccinate. AI chatbots may be a helpful tool as an adjunct to public health campaigns in eliminating vaccine hesitancy. Future work should include the development of standardized evaluation frameworks for AI tools in health communication. Performance in real-world settings, including evaluating its performance in other languages, would also be an important subject of future study. As newer models are developed, their performance also should be constantly re-evaluated, as these models may be able to overcome the limitations of older models and may be able to provide more personalised and accurate responses.

## Figures and Tables

**Table 1:** Evaluation of ChatGPT responses to commonly asked questions on vaccine hesitancy.

Question	Summary of ChatGPT Response	Evaluation
*Doubting the efficacy of vaccines*		
I think that big pharmaceutical companies are just trying to profit off us. Their vaccines cannot be trusted. Is that correct?	“The idea that pharmaceutical companies are primarily motivated by profit is a common concern, and there are certainly cases where companies prioritize financial gain over other considerations. However, it’s important to understand that vaccine development and production involve a complex mix of motivations, including public health, scientific advancement, and profitability…”	Overall, ChatGPT highlights important reasons for vaccination, benefiting both the individual and society at large, whilst emphasizing that the vaccine is safe. However, the response provided is generic and not tailored to an individual’s specific situation (for example, how likely is a child to travel to an area with higher risk for measles transmission?). Specific to HPV vaccination, ChatGPT appropriately expounds on the positive benefits of HPV vaccination in men, their sexual partners, and society as a whole in reducing HPV transmission and its associated diseases. However, does not acknowledge the age cut-offs suggested by ACIP for vaccination, or take into account an individual’s sexual health and risk for shared decision-making.
My immune system can handle an infection, and I can also choose to avoid crowds. Why would I still need the COVID-19 vaccine?	“While it’s true that some people may have a robust immune system that can handle infections, and taking precautions like avoiding crowds can reduce your risk of exposure, there are several important reasons to consider getting the COVID-19 vaccine…Protection from severe illness… Reducing transmission…”	
Why do I need to give my child the measles vaccine if it is so rare nowadays?	“Even though measles has become relatively rare… it’s still essential to vaccinate your child… for several important reasons… Herd immunity… resurgence risk… serious health consequences… measles can lead to severe complications… the vaccine is safe and effective… public health… international travel… unvaccinated travelers can bring the virus back to their home country.”	
I am a 28-year-old man. The HPV vaccination prevents cervical cancer. Why should a man like me get the HPV vaccine?	“The HPV vaccine is often associated with preventing cervical cancer in women…important benefits for men as well…protection against…anal, penile and oropharyngeal cancers…preventing transmission…to their sexual partners… herd immunity… reducing… genital warts… provides long-lasting protection… discuss with healthcare provider…”	
Isn’t natural immunity better than vaccination to protect against infections?	“Natural immunity, acquired through exposure to an infectious agent, can provide protection against future infections. However, the effectiveness and safety of natural immunity can vary significantly depending on the specific disease and individual factors…. Risk of severe disease… Consistency of protection…”	
*Concerns about the adverse effects of vaccines*		
Will the MMR vaccine cause autism in my child?	“No, the MMR (measles, mumps, and rubella) vaccine does not cause autism. This myth originated from a flawed and widely discredited study published in 1998 by Andrew Wakefield, which falsely claimed a link between the MMR vaccine and autism. The study was based on a small sample size and had significant methodological issues…”	Advice provided is accurate and up-to-date. With regards to MMR vaccination, ChatGPT unequivocally denies the link with autism, which is the consensus of the scientific community. ChatGPT also highlights that newer influenza vaccines no longer contain egg proteins, which had previously been a concern for patients with severe egg allergies. Rather than being dismissive of the concern, ChatGPT gives the context for these concerns and then provides reassurance. It also consistently prompts the individual to discuss further with a healthcare provider. Pertaining to concerns regarding mRNA vaccines, ChatGPT provides a measured response and explains the mechanism with which mRNA vaccines work, in particular highlighting its safety profile. It does not offer non-mRNA vaccine options. It takes the opportunity to encourage and emphasize the importance of COVID-19 vaccination and prompts the individual to discuss further with a healthcare provider. With regards to vaccination in children, ChatGPT addresses parental concerns with regards to long-term safety of vaccines. While ChatGPT acknowledges the uncertainty of this, it reassures the individual that the benefits likely outweigh the risks of vaccination, along with the added reassurance that comes from continued monitoring and safeguards in place with regards to vaccine safety.
I have an egg allergy. I heard that this means I should not take the flu vaccine. Is that true?	“Having an egg allergy doesn’t necessarily mean you can’t receive the flu vaccine… traditional influenza vaccines were manufactured using chicken eggs… however, many flu vaccines have been reformulated to reduce or eliminate the risk of an allergic reaction in people with egg allergies… consult with a health care provider… egg-free vaccines… FluMist nasal spray…in many cases, individuals with egg allergies can still receive flu vaccines safely.”	
I heard that the COVID-19 vaccine will permanently alter my DNA.	“The idea that COVID-19 vaccines can alter your DNA is a misconception that is not supported by the scientific evidence… mRNA vaccines provide temporary instructions to your cell… vaccine components are broken down and eliminated from your body… mRNA… is fragile and rapidly degrades within your cells… DNA remains unchanged… safe and effective in preventing COVID-19 and its severe consequences.”	
If I let my child get a COVID-19 vaccine, how can we be sure that it will not cause long-term side effects decades later?	“Long-term side effects from vaccines are typically extremely rare… high degree of confidence in the safety of vaccines… Extensive Clinical Trials… Regulatory approval… Ongoing monitoring… historical safety record… impossible to guarantee absence of any long-term side effects with absolute certainty… available evidence and safeguards in place provide a high level of confidence in the safety of COVID-19 vaccines.”	
I heard that the COVID-19 vaccine can cause severe complications like myocarditis. Should I take the COVID-19 vaccine?	“The COVID-19 vaccines have been extensively studied, and they have been found to be safe and effective in preventing COVID-19, a disease that can have serious and even life-threatening complications. While it is true that there have been rare reports of myocarditis (inflammation of the heart muscle) and pericarditis (inflammation of the lining around the heart) occurring after COVID-19 vaccination, it’s important to understand the context of these cases…Rare occurrence… Benefits outweigh risks…”	
Can I get an infection from any kind of vaccine?	“Vaccines are designed to stimulate your immune system to produce an immune response without causing the actual disease. In most cases, vaccines do not contain live, active viruses or bacteria that can cause the disease they are intended to prevent. As a result, you generally cannot get the disease from the vaccine itself…”	
*Cultural concerns pertaining to vaccinations*		
Were the COVID-19 vaccines tested on human embryos?	“The COVID-19 vaccines authorized for use in many countries, such as the Pfizer-BioNTech and Moderna vaccines, as well as the Johnson & Johnson vaccine, were not tested on human embryos. These vaccines were developed and tested using well-established scientific and ethical principles…”	Pertaining to ethical and cultural issues, as stated, the responses that ChatGPT provided were factual and maintained objectivity. Importantly, the responses were also measured and were able to acknowledge an individual’s struggle with the issue. However, because details of the religious concern and specific vaccine details were not given, more detailed discussion on the risks and benefits could not be provided by ChatGPT.
My religion doesn’t allow me to get vaccinated. Is it okay if I choose not to take any vaccines?	“The decision to get vaccinated can be a complex one…key points to consider… public health… religious exemptions… consult with religious leaders… Individual and community impact… informed decision… important to be well-informed about the potential risks and benefits of vaccines and consult with healthcare professionals…”	
It is important for me to start a family. Will the COVID-19 vaccine affect my fertility?	“There is no credible scientific evidence to suggest that the COVID-19 vaccines have a negative impact on fertility. The COVID-19 vaccines authorized for use have undergone rigorous clinical trials and have been closely monitored for safety…”	
Were any animals harmed in the production of vaccines?	“The development and production of vaccines typically involve preclinical testing on animals to ensure their safety and effectiveness before human clinical trials. This is a common practice in pharmaceutical research to better understand how a vaccine or drug may affect living organisms. However, it’s essential to note that there are strict ethical guidelines and regulations in place to minimize harm to animals in research…”	

## Data Availability

Data may be made available on reasonable request from the corresponding author.

## Abbreviations

The following abbreviations are used in this manuscript:

ACIP	Advisory Committee on Immunization Practices
AI	Artificial intelligence
COVID-19	Coronavirus disease 2019
DNA	Deoxyribonucleic acid
HPV	Human Papillomavirus
MMR	Measles, mumps, and rubella
mRNA	Messenger ribonucleic acid

## Appendix A

**Table A1:** ChatGPT responses to questions pertaining to vaccine hesitancy.

Questions and Responses
*Doubting the efficacy of vaccines* **Q: I think that big pharmaceutical companies are just trying to profit off us. Their vaccines cannot be trusted. Is that correct?** A: The idea that pharmaceutical companies are primarily motivated by profit is a common concern, and there are certainly cases where companies prioritize financial gain over other considerations. However, it’s important to understand that vaccine development and production involve a complex mix of motivations, including public health, scientific advancement, and profitability. Here are a few key points to consider: 1. **Regulatory Oversight:** Vaccines are subject to rigorous testing and evaluation by regulatory agencies like the FDA in the United States and the European Medicines Agency (EMA) in Europe. These agencies have strict safety and efficacy standards that vaccines must meet before they are approved for use. This oversight is designed to protect public health. 2. **Public Health Benefit:** Vaccines have been instrumental in reducing or even eradicating many serious diseases, such as smallpox and polio. The primary goal of vaccination is to protect the public from these diseases and prevent outbreaks. 3. **Research and Development Costs:** Developing vaccines is a costly and risky endeavor, involving extensive research, clinical trials, and regulatory processes. Companies do expect to recover their costs and make a profit, but that doesn’t necessarily mean their products are ineffective or unsafe. 4. **Transparency and Accountability:** There is scrutiny and transparency throughout the vaccine development process. Research results are subject to peer review, and any adverse events or safety concerns are reported and investigated. It’s essential to critically evaluate the information and sources you rely on when forming an opinion about vaccines. There’s no doubt that vaccines have saved countless lives and prevented significant suffering, but like any medical intervention, there can be risks and side effects. If you have concerns about a specific vaccine or vaccines in general, it’s a good idea to consult with healthcare professionals and look at information from reputable sources such as government health agencies and reputable medical organizations. In summary, while profit is a factor in the pharmaceutical industry, vaccines are subject to strict regulation and have a significant public health benefit. It’s crucial to make informed decisions about vaccination based on the available scientific evidence and recommendations from healthcare experts. **Q: My immune system can handle an infection, and I can also choose to avoid crowds. Why would I still need the COVID-19 vaccine?** A: While it’s true that some people may have a robust immune system that can handle infections, and taking precautions like avoiding crowds can reduce your risk of exposure, there are several important reasons to consider getting the COVID-19 vaccine: 1. **Protection from Severe Illness:** COVID-19 can cause a wide range of symptoms, from mild to severe, and in some cases, it can lead to hospitalization, long-term complications, or even death. The vaccine significantly reduces the risk of severe illness and hospitalization if you do contract the virus. 2. **Reducing Transmission:** Getting vaccinated helps reduce the spread of the virus within the community. Even if you have a mild or asymptomatic case, you can still spread the virus to others, including those who may be more vulnerable to severe illness. 3. **Variants:** New variants of the virus continue to emerge, and some may be more transmissible or potentially more resistant to immunity developed through previous infection. Vaccines are designed to be effective against these variants and provide a level of protection that may not be achieved through natural infection alone. 4. **Herd Immunity:** Widespread vaccination is a critical component of achieving herd immunity, which is the point at which enough of the population is immune to the virus, making it difficult for the virus to spread. This protects even those who cannot receive the vaccine due to medical reasons or age. 5. **Long-Term Effects:** While some individuals experience lingering symptoms or “long COVID” even after a mild infection, the long-term effects of COVID-19 are still being studied. Vaccination can help reduce the risk of these potential long-term complications. 6. **Travel and Public Activities:** Many countries and regions require proof of vaccination for travel or entry to certain events and activities. Getting vaccinated may allow you to participate in these without restrictions. 7. **Global Efforts:** The global community’s efforts to control the pandemic and protect vulnerable populations depend on widespread vaccination. Being part of this collective effort is a responsible and ethical choice. Ultimately, the decision to get vaccinated against COVID-19 is a personal one, but it’s essential to weigh the potential benefits and risks, consider the advice of healthcare experts and authorities, and think about the broader impact on public health. Discussing your concerns and questions with a healthcare provider can also be helpful in making an informed decision. **Q: I am a 28-year-old man. The HPV vaccination prevents cervical cancer. Why should a man like me get the HPV vaccine?** A: The HPV (human papillomavirus) vaccine is often associated with preventing cervical cancer in women, but it’s important to note that the vaccine offers important benefits for men as well. Here are several reasons why a man like you may want to consider getting the HPV vaccine: 1. **Protection Against HPV-Related Cancers:** HPV is not only a risk factor for cervical cancer but also for other types of cancer, including anal, penile, and oropharyngeal (throat) cancers. Men can develop these cancers, and the HPV vaccine can help reduce the risk. 2. **Preventing Transmission:** Men can carry and transmit HPV to their sexual partners. By getting vaccinated, you not only protect yourself but also contribute to reducing the overall prevalence of the virus, which benefits public health and helps protect your sexual partners. 3. **Herd Immunity:** Widespread vaccination in both men and women helps achieve herd immunity, making it more challenging for the virus to spread in the community. This offers protection even to those who may not be able to receive the vaccine. 4. **Increased Sexual Health:** By reducing the risk of HPV-related cancers and genital warts (another condition caused by HPV), the vaccine can contribute to better sexual health and well-being. 5. **Lifetime Protection:** HPV vaccination provides long-lasting protection. Getting vaccinated while you’re young can help ensure you’re protected for a lifetime. It’s important to discuss HPV vaccination with your healthcare provider to determine the most appropriate timing and vaccine type for your specific situation. While the vaccine is most effective when administered before sexual activity begins, it can still be beneficial for individuals who have already become sexually active. Making an informed decision about vaccination can help protect your health and the health of your partners. **Q: Isn’t natural immunity better than vaccination to protect against infections?** A: Natural immunity, acquired through exposure to an infectious agent, can provide protection against future infections. However, the effectiveness and safety of natural immunity can vary significantly depending on the specific disease and individual factors. When it comes to infectious diseases, whether natural immunity is better than vaccination depends on several important considerations: 1. **Risk of Severe Disease:** Some infections can lead to severe illness, long-term complications, or even death. Relying on natural immunity to acquire protection means risking exposure to the disease and the potential for these serious consequences. Vaccination offers a safer way to achieve immunity without experiencing the full disease. 2. **Consistency of Protection:** The level and duration of immunity acquired through natural infection can vary widely. In some cases, natural immunity may be robust and long-lasting, but for other diseases, it may wane over time. Vaccines are designed to provide consistent and durable protection. 3. **Reduced Risk of Transmission:** Vaccination not only protects the individual but also contributes to reducing the spread of diseases in the community. Achieving herd immunity through vaccination can protect those who cannot be vaccinated due to medical reasons or age. 4. **Controlled Exposure:** Vaccines are formulated to provide a controlled and weakened form of the infectious agent, reducing the risk of severe disease while still allowing the immune system to develop a strong and specific response. Natural infection can lead to uncontrolled exposure, which may result in more severe illness. 5. **Safety:** Vaccines undergo rigorous testing and safety evaluations before approval. While vaccines can have side effects, they are generally safer than the full-blown disease. Natural infection can result in complications and adverse outcomes. 6. **Variants:** Natural immunity may be less effective against new variants of a virus, while vaccines can be adapted to offer better protection against emerging strains. In summary, while natural immunity can be effective for some diseases, vaccines are generally a safer, more reliable, and more controlled way to achieve immunity, especially for diseases with severe consequences. Vaccination plays a critical role in public health by reducing the spread of infectious diseases and protecting both individuals and communities. Decisions about vaccination should be made based on the best available scientific evidence and guidance from healthcare professionals and public health authorities. **Q: Why do I need to give my child the measles vaccine if it is so rare nowadays?** A: Even though measles has become relatively rare in many parts of the world, it’s still essential to vaccinate your child against it for several important reasons: 1. **Herd Immunity:** Measles is highly contagious, and outbreaks can occur when a significant portion of the population is not immune. By vaccinating your child, you contribute to achieving and maintaining herd immunity. This protects those who cannot receive the vaccine due to medical reasons, such as severe allergies or weakened immune systems, as well as those who are too young to be vaccinated. 2. **Resurgence Risk:** While measles may be rare in some regions, it has not been eradicated globally. Travel and the movement of people can reintroduce the virus to areas where it was previously controlled. Vaccination helps prevent the resurgence of measles. 3. **Serious Health Consequences:** Measles can lead to severe complications, including pneumonia, encephalitis (inflammation of the brain), and death. Vaccination is the most effective way to prevent these serious health consequences. 4. **Vaccine is Safe and Effective:** The measles vaccine is highly effective and safe. It has been widely used for decades and has saved countless lives. 5. **Public Health:** Protecting the health of the community is a collective responsibility. Measles vaccination not only safeguards your child’s health but also contributes to the well-being of society as a whole. 6. **International Travel:** If you plan to travel internationally, vaccination is especially important. Measles remains common in many parts of the world, and unvaccinated travelers can bring the virus back to their home country. Vaccination against measles is a crucial component of public health efforts to prevent the spread of preventable diseases. While the disease may seem rare, it is not eliminated, and the best way to keep it that way is to ensure high vaccine coverage to maintain herd immunity and protect vulnerable individuals.
*Concerns about the adverse effects of vaccines* **Q: Will the MMR vaccine cause autism in my child?** A: No, the MMR (measles, mumps, and rubella) vaccine does not cause autism. This myth originated from a flawed and widely discredited study published in 1998 by Andrew Wakefield, which falsely claimed a link between the MMR vaccine and autism. The study was based on a small sample size and had significant methodological issues. Subsequent extensive research, including large-scale epidemiological studies, has consistently shown no connection between the MMR vaccine and autism. The original study was retracted, and Andrew Wakefield lost his medical license due to ethical violations and conflicts of interest. The scientific consensus is that vaccines, including the MMR vaccine, are safe and highly effective at preventing potentially serious and life-threatening diseases. Fears of a connection between vaccines and autism have been thoroughly investigated and debunked. Vaccination is a crucial part of public health efforts to prevent the spread of infectious diseases and protect individuals and communities. It’s important to rely on reputable scientific and medical sources for information about vaccines and their safety. **Q: I have an egg allergy. I heard that this means I should not take the flu vaccine. Is that true?** A: Having an egg allergy doesn’t necessarily mean you can’t receive the flu vaccine. In the past, some flu vaccines, particularly the traditional influenza vaccines, were manufactured using chicken eggs, which could potentially contain traces of egg proteins. This led to concerns about administering these vaccines to individuals with egg allergies. However, many flu vaccines have been reformulated to reduce or eliminate the risk of an allergic reaction in people with egg allergies. Some flu vaccines are now considered safe for individuals with egg allergies, even those with a history of severe allergic reactions to eggs. The guidance can vary depending on the type of flu vaccine, so it’s important to consult with a healthcare provider who can recommend the most appropriate vaccine for your specific situation. In general, the following guidelines can help: 1. **Consult with a Healthcare Provider:** If you have an egg allergy, it’s essential to discuss your specific allergy history with a healthcare provider. They can provide guidance on which flu vaccine is suitable for you. 2. **Egg-Free Vaccines:** Some flu vaccines are explicitly labeled as “egg-free” or suitable for people with egg allergies. These vaccines are often recommended for individuals with egg allergies. 3. **FluMist Nasal Spray:** If you have an egg allergy and are eligible for the nasal spray flu vaccine (FluMist), this can be an option, as it is egg-free. 4. **Vaccine Administration:** If you do receive a flu vaccine, it’s advisable to do so in a healthcare setting where trained medical personnel can respond to any allergic reactions promptly. Ultimately, the most important thing is to work with a healthcare professional who can consider your specific medical history and provide guidance on the safest and most appropriate flu vaccine for you. In many cases, individuals with egg allergies can still receive flu vaccines safely. **Q: I heard that the COVID-19 vaccine can cause severe complications like myocarditis. Should I take the COVID-19 vaccine?** A: The COVID-19 vaccines have been extensively studied, and they have been found to be safe and effective in preventing COVID-19, a disease that can have serious and even life-threatening complications. While it is true that there have been rare reports of myocarditis (inflammation of the heart muscle) and pericarditis (inflammation of the lining around the heart) occurring after COVID-19 vaccination, it’s important to understand the context of these cases. 1. **Rare Occurrence:** Myocarditis and pericarditis after COVID-19 vaccination are rare, and the cases reported have been mostly mild, especially among young adults and adolescents. The benefits of vaccination in preventing COVID-19 outweigh the risks of these rare side effects. 2. **Benefits Outweigh Risks:** COVID-19 itself can lead to severe complications, including heart issues, and has caused millions of deaths worldwide. Vaccination significantly reduces the risk of severe illness, hospitalization, and death from COVID-19. 3. **Monitoring and Management:** Health authorities closely monitor the safety of vaccines, and the cases of myocarditis and pericarditis are being investigated. It’s important to note that healthcare providers can effectively manage these conditions when they occur. 4. **Individual Risk Assessment:** Your decision to get vaccinated should take into account your individual health situation and potential risks. If you have concerns about the vaccine’s potential side effects, it’s advisable to discuss them with a healthcare provider who can provide guidance based on your specific circumstances. In general, the overwhelming consensus among healthcare experts is that the benefits of COVID-19 vaccination far outweigh the potential risks, especially given the severe health consequences of COVID-19 itself. The vaccines have been critical tools in controlling the spread of the virus and protecting public health. It’s important to make an informed decision based on the advice of healthcare professionals and reputable health agencies. **Q: I heard that the COVID-19 vaccine will permanently alter my DNA.** A: The idea that COVID-19 vaccines can alter your DNA is a misconception and not supported by scientific evidence. COVID-19 vaccines, such as the Pfizer-BioNTech and Moderna vaccines, are based on messenger RNA (mRNA) technology, and the Johnson & Johnson vaccine is based on viral vector technology. These vaccines work by instructing your cells to produce a harmless piece of the spike protein found on the surface of the coronavirus. Your immune system then recognizes this protein and creates an immune response. Here are some key points to understand: 1. **Temporary Instructions:** The mRNA or viral vector in the vaccine provides temporary instructions to your cells to produce a specific protein. It does not integrate into your DNA or alter your genetic code. Once the protein is produced, the vaccine components are broken down and eliminated from your body. 2. **Rapid Degradation:** The mRNA in the vaccine is fragile and rapidly degrades within your cells. It does not linger or become a permanent part of your genetic material. 3. **DNA Remains Unchanged:** Your DNA, which is located in the cell nucleus, remains unaffected by the COVID-19 vaccine. The vaccine works in the cell cytoplasm, which is a different part of the cell. 4. **Safe and Effective:** These COVID-19 vaccines have undergone rigorous testing in clinical trials and have been authorized for emergency use or fully approved by regulatory agencies based on their safety and efficacy. The concerns about vaccines altering DNA often stem from a misunderstanding of how the technology works. COVID-19 vaccines are a critical tool in controlling the spread of the virus and have been shown to be safe and effective in preventing COVID-19 and its severe consequences. It’s important to rely on information from reputable health authorities and consult with healthcare professionals if you have questions or concerns about the COVID-19 vaccines. **Q: Can I get an infection from any kind of vaccine?** A: Vaccines are designed to stimulate your immune system to produce an immune response without causing the actual disease. In most cases, vaccines do not contain live, active viruses or bacteria that can cause the disease they are intended to prevent. As a result, you generally cannot get the disease from the vaccine itself. However, vaccines can cause mild and temporary side effects. These side effects are typically a sign that your immune system is responding to the vaccine, and they may include: 1. **Soreness at the Injection Site:** This is common with many vaccines and is usually mild. 2. **Fever:** A mild fever may occur after some vaccines as a sign of the body’s immune response. 3. **Fatigue:** Feeling tired or fatigued is a common side effect of many vaccines. 4. **Headache:** Some vaccines can cause mild headaches. These side effects are generally short-lived and resolve on their own. They are typically much milder than the symptoms of the actual disease, and they are a sign that the vaccine is working to build immunity. In rare cases, vaccines can cause more serious side effects, but these are usually outweighed by the benefits of vaccination in preventing potentially severe diseases. The safety and efficacy of vaccines are rigorously tested in clinical trials before they are authorized or approved for use. Public health agencies closely monitor vaccines’ safety through systems like the Vaccine Adverse Event Reporting System (VAERS) to ensure their ongoing safety. It’s important to discuss any concerns you have about vaccines with your healthcare provider, as they can provide information specific to the vaccine you’re considering and your individual health circumstances. **Q: If I let my child get a COVID-19 vaccine, how can we be sure that it will not cause long-term side effects decades later?** A: Long-term side effects from vaccines are typically extremely rare, and they are closely monitored through ongoing surveillance systems. While it is challenging to predict the possibility of side effects decades later with absolute certainty, the following factors provide a high degree of confidence in the safety of vaccines, including COVID-19 vaccines: 1. **Extensive Clinical Trials:** COVID-19 vaccines underwent rigorous clinical trials that involved tens of thousands of participants. These trials provided essential data on the safety and effectiveness of the vaccines. 2. **Regulatory Approval:** Vaccines are subject to review and approval by regulatory agencies, such as the U.S. Food and Drug Administration (FDA) and the European Medicines Agency (EMA). These agencies assess the data from clinical trials and review the safety profiles of vaccines before granting authorization or approval. 3. **Ongoing Monitoring:** Even after approval, vaccines are continuously monitored for safety. Health authorities use systems like the Vaccine Adverse Event Reporting System (VAERS) to track and investigate any reported adverse events. This ongoing surveillance is designed to detect any rare or unexpected long-term side effects. 4. **Historical Safety Record:** Vaccines have an excellent track record of safety. Many well-established vaccines have been in use for decades without the emergence of unexpected long-term side effects. 5. **Scientific Understanding:** The scientific principles behind vaccine development and how the immune system responds to vaccines are well-understood. This knowledge informs vaccine design and minimizes potential risks. 6. **Global Collaboration:** COVID-19 vaccines have been developed through unprecedented international collaboration, drawing on the expertise of scientists and researchers worldwide. 7. **Ethical Considerations:** The ethical standards governing vaccine development and testing are designed to minimize risks to participants in clinical trials and prioritize safety. While it is impossible to guarantee the absence of any long-term side effects with absolute certainty, the available evidence and safeguards in place provide a high level of confidence in the safety of COVID-19 vaccines. The known risks of COVID-19, including severe illness, hospitalization, and death, make the potential benefits of vaccination outweigh the risks for most people. Ultimately, the decision to vaccinate your child should be based on the best available scientific evidence, guidance from healthcare professionals, and consideration of the risks and benefits. Discuss your concerns with your child’s healthcare provider to make an informed choice for their health and well-being.
*Cultural concerns pertaining to vaccines* **Q: My religion doesn’t allow me to get vaccinated. Is it okay if I choose not to take any vaccines?** A: The decision to get vaccinated can be a complex one, and it may be influenced by various factors, including personal beliefs and religious convictions. However, it’s important to consider the potential impact of your decision on public health and the community, especially when it comes to vaccines that prevent contagious diseases. Here are some key points to consider: 1. **Public Health:** Vaccination is not only a personal health choice but also a matter of public health. Herd immunity, which is achieved when a significant portion of a population is immune to a contagious disease, helps protect those who cannot receive vaccines due to medical reasons or age. By choosing not to vaccinate, you may indirectly put vulnerable individuals at risk. 2. **Religious Exemptions:** Many countries and states allow for religious exemptions from vaccination requirements. If your religion prohibits vaccination, you may be eligible for an exemption. However, it’s essential to be aware of the specific laws and regulations in your area and follow any legal requirements for obtaining an exemption. 3. **Consult with Religious Leaders:** If you have concerns about vaccination based on religious beliefs, it can be valuable to consult with religious leaders or scholars within your faith community. They may provide guidance or insights that can help you make an informed decision aligned with your religious values. 4. **Individual and Community Impact:** Consider the potential consequences of your decision on both your individual health and the health of the community. Weigh the benefits of vaccination against the risks of contracting and spreading preventable diseases. 5. **Informed Decision:** Regardless of your decision, it’s important to be well-informed about the potential risks and benefits of vaccines and consult with healthcare professionals to understand the medical and public health implications. It’s important to remember that vaccines have played a significant role in reducing the incidence of serious and potentially deadly diseases. When making a decision about vaccination, you should carefully consider the potential consequences, consult with knowledgeable individuals in your faith community, and abide by the laws and regulations in your jurisdiction. **Q: Were the COVID-19 vaccines tested on human embryos?** A: The COVID-19 vaccines authorized for use in many countries, such as the Pfizer-BioNTech and Moderna vaccines, as well as the Johnson & Johnson vaccine, were not tested on human embryos. These vaccines were developed and tested using well-established scientific and ethical principles. Vaccine development typically involves preclinical testing on animals and then clinical trials in humans. The clinical trials include phases where the vaccines are tested on thousands of human volunteers to assess their safety and effectiveness. These trials follow strict ethical guidelines and regulatory standards to ensure the safety of participants. It’s important to note that the Pfizer-BioNTech and Moderna vaccines are based on messenger RNA (mRNA) technology, and the Johnson & Johnson vaccine is based on viral vector technology. None of these vaccines involved the use of human embryos in their development or testing. If you have specific concerns or questions about vaccine development and testing, it’s advisable to consult with healthcare professionals and refer to reputable sources for accurate information about the vaccines and the methods used in their development. **Q: it is important for me to start a family. Will the COVID-19 vaccine affect my fertility?** A: There is no credible scientific evidence to suggest that the COVID-19 vaccines have a negative impact on fertility. The COVID-19 vaccines authorized for use have undergone rigorous clinical trials and have been closely monitored for safety. Claims of COVID-19 vaccines affecting fertility have been widely debunked, and these claims are not supported by scientific research. In fact, several medical organizations, including the American College of Obstetricians and Gynecologists (ACOG) and the Centers for Disease Control and Prevention (CDC), recommend COVID-19 vaccination for individuals who are trying to conceive, pregnant, or breastfeeding. It’s important to understand that the risks of COVID-19 infection during pregnancy can be serious, and vaccination can provide protection for both you and your future family. If you have concerns about the vaccine and fertility, it’s advisable to discuss them with a healthcare provider who can provide information specific to your situation and address any questions you may have. Starting a family is a significant decision, and it’s essential to make informed choices regarding your health and well-being. **Q: Were any animals harmed in the production of vaccines?** A: The development and production of vaccines typically involve preclinical testing on animals to ensure their safety and effectiveness before human clinical trials. This is a common practice in pharmaceutical research to better understand how a vaccine or drug may affect living organisms. However, it’s essential to note that there are strict ethical guidelines and regulations in place to minimize harm to animals in research. Animal studies are conducted with care and oversight to protect the welfare of the animals involved. Researchers work to use the smallest number of animals possible and employ methods that cause the least amount of harm or distress. The goal of such animal testing is to determine the safety and efficacy of vaccines, and it plays a critical role in the development of vaccines that protect human health. It is important to strike a balance between advancing medical knowledge and ensuring animal welfare, and regulatory agencies, ethical review boards, and research institutions are responsible for overseeing these practices. It’s important to keep in mind that animal testing is a standard and necessary part of vaccine and drug development, but it is regulated to minimize harm and ensure the safety and effectiveness of medical products. This testing helps provide a foundation of knowledge for the development of vaccines that have saved countless human lives.
